# The power of microRNAs as diagnostic and prognostic biomarkers in liquid biopsies

**DOI:** 10.20517/cdr.2019.103

**Published:** 2020-02-21

**Authors:** Lorena Quirico, Francesca Orso

**Affiliations:** ^1^Department of Molecular Biotechnology and Health Sciences, University of Torino, Torino 10126, Italy.; ^2^Molecular Biotechnology Center (MBC), University of Torino, Torino 10126, Italy; ^3^Center for Complex Systems in Molecular Biology and Medicine, University of Torino, Torino 10126, Italy.

**Keywords:** MicroRNAs, liquid biopsies, diagnosis, prognosis, therapy

## Abstract

In the last decades, progresses in medical oncology have ameliorated the treatment of patients and their outcome. However, further improvements are still necessary, in particular for certain types of tumors such as pancreatic, gastric, and lung cancer as well as acute myeloid leukemia where early detection and monitoring of the disease are crucial for final patient outcome. Liquid biopsy represents a great advance in the field because it is less invasive, less time-consuming, and safer compared to classical biopsies and it can be useful to monitor the evolution of the disease as well as the response of patients to therapy. Liquid biopsy allows the detection of circulating tumor cells, nucleic acids, and exosomes not only in blood but also in different biological fluids: urine, saliva, pleural effusions, cerebrospinal fluid, and stool. Among the potential biomarkers detectable in liquid biopsies, microRNAs (miRNAs) are gaining more and more attention, since they are easily detectable, quite stable in biological fluids, and show high sensitivity. Many data demonstrate that miRNAs alone or in combination with other biomarkers could improve the diagnostic and prognostic power for many different tumors. Despite this, standardization of methods, sample preparation, and analysis remain challenging and a huge effort should be made to address these issues before miRNA biomarkers can enter the clinic. This review summarizes the main findings in the field of circulating miRNAs in both solid and hematological tumors.

## Introduction

In the last years, cancer research has focused on understanding the basis of tumor development as well as looking for new biomarkers and techniques to improve cancer prevention and early detection, as well as monitor disease progression and response to therapies. A milestone in this second goal of cancer research was the development of liquid biopsies that are less invasive, less time-consuming, and safer compared to classical biopsies that are still the gold standard for cancer diagnosis. Liquid biopsies are becoming increasingly precise and standardized. Among the biomarkers found in liquid biopsy specimens, nucleic acids together with proteins are potentially optimal candidates as biomarkers and can be easily isolated from different body fluids. Besides the circulating DNA, circulating RNAs are acquiring relevance as new potential biomarkers because RNA detection is more sensitive, more specific, and cheaper than classical protein biomarkers. At the same time, they can give better dynamic insights of the cell status if compared to circulating DNA. Moreover, the discovery of different classes of regulatory RNAs encoded by the so-called “junk DNA”, namely microRNAs (miRNAs), circular RNAs (circRNAs), ribosomal RNAs (rRNA), transfer RNAs (tRNAs), Piwi-interacting RNAs (piRNAs), and long non-coding RNAs (lncRNAs), has increased the interest in the potential use of these regulatory molecules as therapeutic targets as well as biomarkers for a variety of diseases. Nevertheless, some specific RNAs, such as miRNAs and circRNAs, are stable in the majority of body fluids. In the present review, we discuss the growing importance of miRNAs as cancer biomarkers in liquid biopsies for the main solid and hematological tumors.

## Liquid biopsy

**Figure 1:**
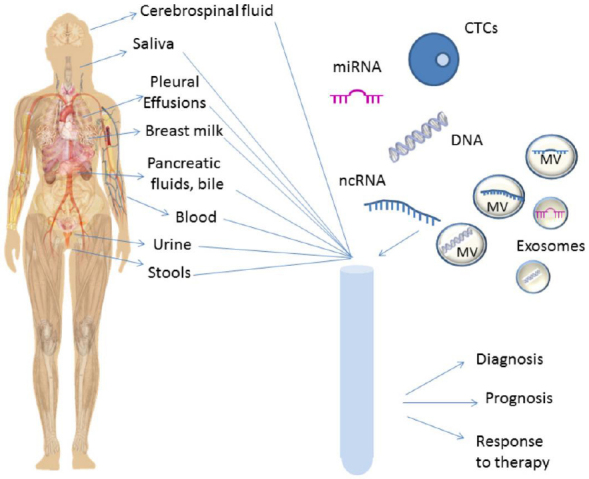
Liquid biopsy. Schematic view of the biological fluids used for liquid biopsy and of the main tumor components analyzed in these fluids. CTC: circulating tumor cell; MV: microvesicle; miRNA: microRNA; ncRNA: non-coding RNA

In the last decades, precision medicine has dramatically renewed medical oncology, introducing patient-tailored therapies with a significant improvement in patient outcome. However, further advancements are necessary, in particular early detection and precise monitoring of the disease are crucial to reduce cancer mortality. Liquid biopsy is a revolutionary technique that allows the detection of Circulating Tumor Cells (CTCs), nucleic acids, and exosomes released by the tumor in the bloodstream and in other biological fluids such as urine^[1]^, saliva^[2]^, pleural effusions^[3]^, cerebrospinal fluid (CSF)^[4]^, and stool^[5]^, as shown in Figure 1. Even if it is not yet a standard procedure in medical oncology, it represents an alternative to classical biopsies that are invasive, risky, and in some cases cannot be performed, e.g., when clinical conditions worsen or when tumor is inaccessible. Moreover, the classical biopsy depicts the tumor status in a precise moment, while tumors evolve over time changing completely their genomic landscape. Liquid biopsy can be used for tumor diagnosis as well as for monitoring tumor recurrence or response to therapy^[6]^. Originally, liquid biopsy was introduced to analyze CTCs and now it is predominantly used to analyze circulating tumor DNA (ctDNA)^[7]^. The low amount of viable CTCs in the circulation and the risk of contamination by DNA from normal blood cells are challenges for this analysis^[8]^. The initial limitation caused by the scarcity of the starting material in the biological fluids has been overcome thanks to the increased sensitivity of next-generation sequencing (NGS) techniques. Even if liquid biopsy for ctDNA expanded enormously in the last five years, the field is evolving rapidly and there is an urgent need for novel tumor biomarkers and detection methods to further improve the power of liquid biopsy.

## miRNAs as potential biomarkers

Recently, biomarker research has focused on RNA molecules circulating in the body fluids. RNA analysis is highly sensitive and specific, cheaper than protein analysis, and offers a more dynamic view of cell regulation and states compared to DNA^[9]^. Long RNA species, however, can be easily degraded by RNAse activity, while shorter RNA molecules, as small non-coding RNAs, are more stable and highly expressed in the blood of cancer patients^[10,11]^.

**Figure 2:**
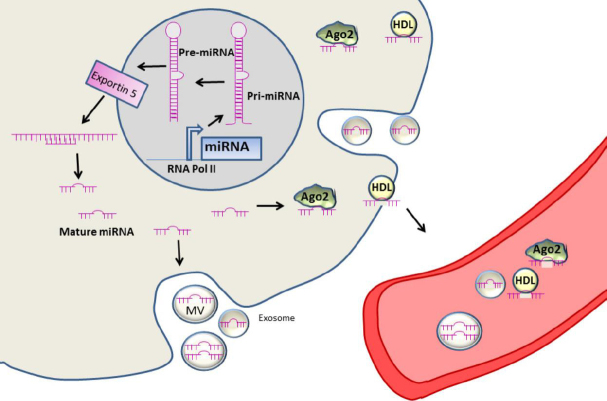
miRNA biogenesis and release. miRNAs are transcribed in the nucleus by RNA pol II into pri-miRNA, which is processed by Drosha into precursor RNA (pre-miRNA) and exported to the cytoplasm where it is processed into mature miRNA and loaded into the miRNA-inducing silencing complex, which is responsible of the production of functional miRNAs. Mature miRNAs can be released in the biological fluids embedded into vesicles (microvesiscles and exosomes) or bound to AGO2 or HDL. MV: microvesicle; miRNA: microRNA; HDL: high density lipoprotein; AGO2: argonaute protein 2

miRNAs are small non-coding RNAs (19-22 nucleotides) originally discovered in *Caenorhabditis elegans* in 1993^[12]^. In the last decades, miRNAs have been described in all animal models, and some of them resulted highly conserved across species^[13,14]^. miRNAs are transcribed by RNA II polymerase into primary miRNAs or pri-miRNAs (30 nucleotides) containing a specific hairpin-shaped stem-loop structure, which is recognized and cleaved by the two RNase III endonucleases, Drosha and Dicer, and the regulatory binding protein DGCR8/Pasha. The product of this cleavage is a pre-miRNA that is transported to the cytoplasm by Exportin-5 and its co-factor Ran-GTP^[15]^. Pre-miRNAs are then converted into double-stranded miRNA duplexes that are 19-24 nucleotides long (miRNA: miRNA*) by the RNAse III endonuclease Dicer. miRNA duplexes are then loaded into the Argonaute family of proteins, giving rise to the miRNA-inducing silencing complex, which is responsible for the production of functional miRNAs^[15]^. In the canonical mechanism of action, the mature miRNA binds to the 3’UTR of target mRNAs and, based on a kind of complementarity, miRNAs can repress translation or induce deadenylation and mRNA decay^[15]^. miRNAs are often deregulated in different diseases and, in particular, they are crucial regulators of cancer onset and progression, behaving as oncogenes or tumor suppressors. Oncogenic miRNAs are overexpressed in tumor cells and they exert their tumor promoting function by targeting tumor suppressor genes. On the contrary, tumor suppressor miRNAs are downregulated or deleted in tumors where they regulate the targets involved in the control of tumor proliferation and survival^[16]^. Moreover, miRNA expression is not only critical in tumor tissues, but also in the circulation. miRNAs can be released into extracellular fluids such as plasma, serum^[17,18]^, CSF^[19]^, saliva^[20]^, breast milk^[21]^, urine^[22]^, ovarian follicular fluid^[23]^, pancreatic fluid, bile^[24]^, peritoneal fluid^[25]^, and stool^[26]^. Nowadays, miRNAs are considered as useful disease biomarkers since they are highly stable in body fluids (up to four days at room temperature) and they are resistant to high or low pH, multiple freeze-thaw cycles, and long-term storage^[11,17]^. In body fluids, extracellular miRNAs are protected against enzymatic degradation thanks to their association with RNA binding proteins (Argonaute-2 and nucleophosmin-1), with high- and low-density lipoproteins, or to their embedding in membrane vesicles such as exosomes, microvescicles, and apoptotic bodies^[18,27,28]^, as shown in Figure 2. Moreover, the quantitation of extracellular miRNAs is easy and highly sensitive due to the introduction of technological platforms (e.g., NGS) that detect nucleic acids with great accuracy^[29]^. Secreted miRNAs act through a hormone-like mechanism for cellular communication, behaving as autocrine, paracrine, and endocrine regulators of cellular functions^[30,31]^. Extracellular miRNAs can be considered as biomarkers for early detection and prognosis of cancer as well as for defining cancer staging and therapeutic outcome. The potential diagnostic and prognostic use of extracellular miRNAs for the main solid and hematological tumors is discussed in the next sections. Due to the high number of studies related to this topic, we give a general overview of the main achievements for the different neoplasia, as summarized in Table 1 for solid tumors and Table 2 for hematological ones.

**Table 1:** miRNAs as diagnostic and prognostic markers in solid tumors

Cancer type	miRNAs	Specimen	Reference	Use
Breast Cancer	miR-155, miR-19a miR-181b, miR-24	Serum	33, 34	D, P, T
	miR-195	Serum	35	D
	miR-10b	Serum	34	D, P
	miR-1246, miR-1307-3p, miR-4634, miR-6861-5p, miR-6875-5p	Serum	36, 37	D
	miR-21	Exosomes	37	D
	miR-329	Serum	38	D, P
	miR-331, miR-195	Serum	39	D, P
	miR-106b	CTCs	40	D, P
	miR-200a, miR-210	Plasma	41	D, P, T
Colorectal Cancer	miR-92a, miR-144*	Stool	45, 47	D
	miR-21	Stool, serum, plasma	46,47, 49	D, P
	miR-378, miR-409-3p, miR-93, miR-7, miR-483-5p, miR-422-5p	Plasma	50, 52	D, P
	miR-186-5p, miR-193-5p, miR-342-3p, miR-106-5p, miR-223a-3p			
	miR-335-5p, miR-186-5p, miR-342-3p			
	miR-23a, miR-301a	Exosomal	53	D
	miR-199b-5p,miR-150-5p, miR-29c-5p, miR-218-5p, miR-99a-3p	Exosomal	25	D
	miR-383-5p, miR-199a-3p, miR-193a-5p, miR-10b-5p, miR-101c-5p			
	miR-20a, miR-21, miR-23	Plasma	54	P
	miR-141	Plasma	55	P
	miR-328-3p, miR-652-3p, miR-342-3p, miR-501-3p	Serum	56	T
Gastric Cancer	miR-221, miR-20a, miR-106b	Plasma	59	D
	miR-425-5p, miR-1180-3p, miR-122-5p, miR-24-3p, miR-4632-5p	Plasma	60	D
	miR-21	Plasma	61	D, P
	miR-20a, miR-1, miR-27a, miR-34, miR-423-5p	Plasma	62	D
	miR-378, miR-371-5p, miR-187	Serum	63	D
	miR-515-3p	Serum	64	D
	miR-141	Plasma	65	D
	miR-376c	Plasma, urine	66	D
	miR-6807-5p, miR-6856-5p	Urine	67	D
	miR-10b, miR-21, miR-223, miR-338, let-7a, miR-30-5p, miR-126	Serum, plasma	68	P
	miR-196a, miR-196b	Serum, plasma	69	D, P
	miR-19b, miR-106a	Exosomes	70	D, P
Glioblastoma	miR-21, miR-128, miR-342-3p	Plasma	71	D, P
	miR-21	Exosomes	72	D
	miR-451	Exosomes	73	D
	miR-21-5p, miR-218-5p, miR-193b-3p, miR-331-3p, miR-374a-5p,	CSF	74	D, P
	miR-548c-3p, miR-520f-3p, miR-27b-3p, miR-30b-3p			
	miR-21, miR-125, miR-222	CSF, blood	75	D
Hepatocellular	miR-21, miR-26a, miR-27a, miR-122, miR-192, miR-223, miR-801	Plasma, serum	79	D
Carcinoma	miR-21	Plasma,serum,feces, tissues	81	D
	miR-206, miR-141-3p, miR-499-3p, miR-1228-5p, miR-199a-5p,	Serum	83	D
	miR-122-5p, miR-192-5p, miR-26a-5p			
	miR-221, miR-222, miR-224, miR-21	Serum	84	D, P
	miR-122	Serum	82	D, P
	miR-1972, miR-193a-5p, miR-214-3p, miR-365a-3p, miR-128,	Serum	85	D, P
	miR-139-5p, miR-382-5p, miR-410, miR-424-5p, miR-101-3p			
	miR-155, miR-96a, miR-99a	Serum	86	D, P
	miR-30a, mR-122, miR-125b, miR-200a, miR-374b, miR-15b,	Plasma	87	T
	miR-107, miR-320, miR-645			
Lung Cancer	miR-92a	Plasma	90	D
	miR-125b, miR-22, miR-15b	Serum	91	D, P
	miR-146a, miR-221, let-7a, miR-155, miR-17-5p, miR-27a, miR106a	Plasma	92	D
	miR-17-5p	Exosomes	93	D
	miR-155, miR-197, miR-182	Plasma	94	D, P, T
	miR-30b, miR-30c, miR-103, miR-122, miR-195, miR-203, miR-221,	Esosomes	95	D
	miR-222			
	let-7b, let-7e, miR-223a-3p, miR-486, miR-181-5p, miR-30a-3p,	Exosomes	96	D, P
	miR-30e-3p, miR-361-5p, miR-10b-5p, miR-15b-5pmiR-320b			
	miR-520f	Serum	97	D, P
	miR-25	Plasma	98, 99	D, P
	miR-test	Serum	100	D, P
Melanoma	miR-134-5p, miR-320a-3p	Plasma	104	D, P
	miR-9-5p, miR-145-5p, miR-150-5p, miR-195-5p, miR-205-5p	Serum	105	D, P
	miR-150-5p, miR-15b-5p, miR-199-3p, miR-193a-3p, miR-524-5p	Plasma	106	D, P
	miR-29c, miR-1280, miR-365, miR-1249, miR-328, miR-422a, miR-30d, miR-17			
	miR-29c-5p, miR-324-3p	Serum	107	D, P
	miR-125b	Exosomes	108	D, P
	miR-532-5p, miR-106b	Exosomes	109	D, P, T
	miR-15, miR-30d, miR-15b, miR-425	Serum	110	D, P
	miR-206	Serum	111	D, P, T
	MELmiR-7: miR-16, miR-211, miR-4487, miR-4706, miR-4731,	Serum	112	D, P
	miR-509-3p, miR-509-5p			
	Mel-38	Plasma	113	D, P, T
	let-7g-5p, miR-497-5p	Exosomes	114	P, T
Ovarian Cancer	miR-106a-5p, let-7d-5p, miR-122-5p, miR-185-5p, miR-99b-5p	Exosomes, plasma	115	D
	miR-93 miR-145 miR-200c	Exosomes	116	D
	miR-21, miR-141, miR-200a, miR-200c, miR-200b, miR-203,	Exosomes	117	D
	miR-205, miR-214			
	miR-148a-3p	Plasma	118	P
	miR-34a, miR-34b, miR-141, miR-200a, miR-200c, miR-203,	Plasma	119	D, P
	miR-429			
	miR-10a-5p, miR-145-5p, miR-205-5p, miR-328-5p, miR-346	Exosomes	120	D, P
Pancreatic Cancer	miR-20a, miR21, miR-24, miR-25, miR-99a, miR-185, miR-191	Serum	123	D, P
	miR-21, miR-155	Exosomes, pancreatic juice	124	D, P
	let-7e-5p, let-7f-5p, miR-103a-3p, miR-181a-5p, miR-151b,	Plasma	125	D, P
	miR-23-3p, miR-320a miR-33a-3p, miR-548d-3p, miR-93			
	miR-182-5p, miR-4732-5p, miR-139-5p, miR-23b-3p	Plasma	126	D, P
	miR-10b, miR-30c, miR-106b, miR-132, miR-155, miR-181a,	Plasma, bile	24	D, P
	miR-181b, miR-196a, miR-212			
Prostate Cancer	miR-141	Serum	11	D
	miR-141, miR-145, miR-155	Blood	127	D, P
	miR-98-5p, miR-152-3p, miR-326, miR-4289	Plasma	128	D
	miR-26a	Plasma	129	D, P
	miR-141, miR-375	Serum	130	D, P
	miR-16, miR-148a, miR-195	Plasma	131	D, P
	miR-20a, miR-21, miR-145, miR-221	Plasma	132	D, P
	miR-106a, miR-130b, miR-106a, miR-222	Plasma	133	D, P
	let-7c, let-7e, let-7i, miR-26a-5p, miR-26b-5p, miR-18b-5p,	Serum	134	D, P
	miR-25b-3p			
	miR-1290, miR-375	Exosomes	135	D,P
	miR-17, miR-20a, miR-20b, miR-106a	Serum	136	D, P
	miR-223, miR-874, miR-1207, miR-24, miR-106a, miR-30c, miR-26b	Serum	137	D, P
	miR-21	Serum	138	D, P, T
	miR-200, miR-17	Plasma, serum	139	T

D: diagnosis; P: prognosis; T: response to therapy. miR-test: miR-92a, miR-484, miR-486-5p, miR-328, miR-191, miR-376a, miR-342-3p, miR-331-3p, miR-30c, miR-28-5p, miR-98, miR-17, miR-26b, miR-374a, miR-30b, miR-26a, miR-142-3p, miR-103, miR-126, let-7a, let-7b, let-7d, miR-32, miR-133b, miR-566, miR-432*, miR-223, miR-29a, miR-148a, miR-142-5p, miR-22, miR-148b, miR-140-5p, miR-139-5p. Mel-38: miR-424-5p, miR-548l, miR-34a-5p, miR-497-5p, miR-299-3p, miR-205-5p, miR-1269a, miR-624-3p, miR-138-5p, miR-1-5p, miR-152-3p, miR-1910-5p, miR-181b-5p, miR-3928-3p, miR-3131, miR-301a-3p, miR-1973, miR-520d-3p, miR-454-3p, miR-548a-5p, miR-548ad-3p, miR-1537-3p, miR-4532, miR-553, miR-764, miR-1302, miR-1258, miR-522-3p, miR-1264, miR-1306-5p, miR-219a-2-3p, miR-431-5p, miR-450a-5p, miR-2682-5p, miR-337-5p, miR-27a-3p, miR-4787-3p, miR-154-5p.

**Table 2:** miRNAs as diagnostic and prognostic markers in hematological malignancies

Cancer type	miRNAs	Specimen	Reference	Use
Acute Myeloid Leukemia	miR-155-3p, miR-181-5p	Serum	143	D, P
	miR-150, miR-342	Plasma	144	D, P
	miR-10-5p	Serum	145	D, P
	miR-210	Serum	146	P
	miR-155	Blood	147	P
	miR-10b	Exosomes	148	D, P
	miR-203	Serum	149	D, P
Acute lymphoblastic Leukemia	miR-511, miR- 222, miR-34a, miR-199a-3p, miR-223, miR-221,	Plasma	152	D
	miR-26a			
	miR-155, miR-126	Plasma	153	P
	miR-125b-1, miR-203	Serum	154	D
Chronic Lymphocytic Leukemia	miR-155	Plasma	156	P, T
	miR-155, miR-150, miR-29a, miR-29b and miR-29c	Exosomes	157	D
	miR-150	Serum	158	P
	miR-29b		159	D, P
Non-Hodgkin Lymphoma	miR-155, miR-210, miR-21	Serum	10	D, P
	miR-15a, miR-16-1, miR-29c, miR-155, miR-34a	Serum	162	D
	miR-130a, miR-125b	Serum	163	P, T
Multiple Myeloma	miR-451, miR-638, miR-720, miR-1246, miR-1308, miR-1915	Serum	164	D
	miR-92a	Plasma	165	D, P, T
	miR-148a, miR-181a, miR-20a, miR-221, miR-625, miR-99b	Blood	166	D, P
	miR-142-5p, miR-660, miR-29a	Serum	167	D
	miR-130a, miR-34a, let-7d, miR-744, let-7e	Serum	168	D, P
	miR-92a, miR-30a, miR-451, miR-720, miR-16, miR-25	Serum	169	D, P
	miR-1207-5p, miR-3656, miR-630, miR-451, miR-92a,	Plasma	170	D
	miR-22, miR-223, miR-19b, miR-720, miR-16, miR-20a			
	miR-483-5p			D, P
	miR-214, miR-135b	Serum	171	D, P
	miR-19a	Serum	172	P
	miR-19a,miR-26a-5p, miR-29c-3p, miR-30b-5p, miR-30c-5p,	Serum	173	T
	miR-331-3p			
	miR-16, miR-17, miR-20a, miR-660, miR-19b, miR-331	Serum	174	D, P, T
	miR-130a	Serum	175	D

D: diagnosis; P: prognosis; T: response to therapy

## miRNAs as diagnostic and prognostic markers in solid tumors

### Breast cancer

CA15.3 and BR27.29 are serum biomarkers to diagnose breast cancer (BC), but they lack sensitivity and the diagnosis of the worst kind of BC, Triple Negative BC, still relies on histological grade, lymph node involvement, and estrogen receptor (ER), progesterone receptor, human epidermal growth factor receptor 2 (HER-2) status. Therefore, finding new biomarkers with better sensitivity is required^[32]^.

Sochor *et al*.^[33]^ identified four oncogenic serum miRNAs, namely miR-155, miR-19a, miR-181b, and miR-24, significantly overexpressed in patients at the diagnosis and whose serum expression decreased after surgery (miR-155, miR-181b, and miR-24) or upon therapy (miR-19a). Moreover, these miRNAs were more abundant in the serum of high-risk patients compared to the low-risk group. Another group demonstrated the relevance of miR-155 serum levels in discriminating primary BC patients from healthy controls, whereas serum levels of miR-155, miR-10b, and miR-34a discriminated metastatic breast cancer from healthy subjects. Moreover, miR-34a levels were significantly higher in patients at advanced tumor stages than patients at early tumor stages^[34]^. The importance of miR-155 was also underlined by Heneghan *et al*.^[35]^, who characterized whole blood samples from preoperative patients with breast cancer, prostate cancer, colon cancer, renal cancer, or melanoma and healthy controls. let-7a, miR-10b, and miR-155 resulted differentially expressed. A significant increase in miR-195 was found to be BC specific and able to distinguish BC from the other tumors studied and from controls.

Shimomura *et al*.^[36]^ characterized a five-miRNA panel (miR-1246, miR-1307-3p, miR-4634, miR-6861-5p, and miR-6875-5p) to diagnose early BC compared to non-BC individuals with high sensitivity and specificity. An independent study demonstrated that miR-1246, together with miR-21, was ubiquitous in human exosomes and enriched in BC patients compared to controls^[37]^.

Li *et al*.^[38]^ showed that miR-329 was downregulated in the serum and tissue of BC patients compared to healthy controls and its downregulation was associated with metastasis to lymph node and Tumor Node Metastases (TNM) stage. In another study, McAnena *et al*.^[39]^ identified circulating miR-331 and miR-195, respectively, upregulated and downmodulated in metastatic luminal A patients compared to patients with local tumor or healthy individuals.

Tan *et al*.^[40]^ demonstrated miR-106b upregulation in CTCs derived from patients with metastatic BC compared to primary tumors and healthy donors. Furthermore, the combination of miR-106b, vimentin, and E-cadherin in CTCs could predict overall survival. Concerning response to therapy, high levels of miR-200a and miR-210 in the plasma of metastatic BC patients was associated with chemotherapy resistance. Moreover, miR-200a correlated with the stage in surgery and high miR-210 levels correlated with liver, lung and brain metastasis^[41]^.

### Colorectal cancer

RAS mutational status is used as a biomarker in colorectal cancer (CRC) to predict Epidermal Growth Factor Receptor (EGFR)-antibody response^[42]^. It also correlates with CRC aggressiveness^[43]^ and chemotherapy response^[44]^. The fecal immunochemical test (FIT) and colonoscopy are recommended to detect CRC, but they have low uptake, thus surgery is still the best option in the case of non-metastatic CRC, and chemotherapy administration depends on the disease stage. New techniques and biomarkers are required to improve CRC diagnosis and prognosis. Blood presence in the stool may be an indicator of the disease, thus Choi *et al*.^[45]^ evaluated stool miRNA levels. miR-92a and miR-144* were differently expressed among CRC and control group showing good specificity and sensitivity in CRC detection. In another study, fecal miR-92a, in combination with miR-21 resulted as a promising biomarker for fecal-based CRC diagnosis^[46]^. miR-21 was also upregulated in stool from CRC patients in a study by Link *et al*.^[47]^. Moreover, miR-21 levels were elevated in patients when CRC was diagnosed, but they were also high several years before the diagnosis in serum^[48]^ and plasma^[49]^. Plasma miR-378, miR-409-3p, miR-93, and miR-7 levels could also distinguish CRC from healthy patients^[50,51]^.

Zanutto *et al*.^[52]^ characterized plasma samples derived from FIT-positive (FIT+) individuals undergoing colonoscopy and they identified specific signatures of plasma circulating miRNAs for each kind of endoscopic lesion: low-grade adenoma (LgA), high-grade adenoma (HgA), and cancer lesion (CL). In detail, signatures composed of six miRNAs were identified for LgA (miR-483-5p, miR-423-5p, miR-186-5p, miR-193a-5p, miR-342-3p, and miR-378) and HgA (miR-106b-5p, miR-483-5p, miR-323a-3p, miR-335-5p, miR-186-5p, and miR-342-3p), in addition to a two-miRNA signature (miR-378 and miR-342-3p) for CL.

From the diagnostic point of view, exosomal miR-23a and miR-301a expression was increased in serum samples of CRC patients compared to normal controls, resulting capable of discriminating patients with tumor from normal individuals^[53]^. Peritoneal lavage might also be a source of Extracellular Vesicles (EV); thus, Roman-Canal *et al*.^[25]^ analyzed miRNA differential expression in the peritoneal lavage from non-cancer and CRC individuals. Two hundred ten miRNAs were dysregulated and the best 10 were miR-199b-5p, miR-150-5p, miR-29c-5p, miR-218-5p, miR-99a-3p, miR-383-5p, miR-199a-3p, miR-193a-5p, miR-10b-5p, and miR-181c-5p.

To evaluate miRNA expression level potential in CRC prognosis and their use as markers of recurrence, Pesta *et al*.^[54]^ quantified Carcinoembryonic Antigen (CEA), Carbohydrate Antigen 19-9 (CA19-9), and miRNAs in pre- and postoperative blood plasma samples of CRC patients and during follow-up. A downmodulation in miR-20a, miR-23a, miR-210, and miR-223a plasma levels was detected after surgery. A statistically significant relation to overall survival was recorded for miR-21, miR-20a, and miR-23a in patients who underwent palliative surgery. Finally, the combination of CEA and CA19-9 with miR-21, miR-20a, and miR-23a could better distinguish patients with favorable and unfavorable outcomes.

miR-141 plasma levels increased in CRC patients with stage IV colon cancer compared to metastasis-free patients and its expression correlated with poor survival^[55]^. A signature composed by miR-328-3p, miR-652-3p, miR-342-3p, and miR-501-3p could predict primary CRC progression^[56]^.

### Gastric cancer

Gastric cancer (GC) diagnosis at an early stage is difficult since its symptoms may be mild or absent, resulting in a seven-month median survival^[57]^. Invasive methodologies such as upper gastrointestinal imaging and upper gastrointestinal endoscopy are used to detect early disease. Serum tumor markers, e.g., CEA, CA19-9, and CA72-4, are employed for diagnosis and prognosis, but they lack early-stage sensitivity^[58]^.

Cai *et al*.^[59]^ found increased plasma levels of miR-221, miR-20a, and miR-106b in GC patients compared to normal controls. Zhu *et al*.^[60]^ identified the combination of miR-425-5p, miR-1180-3p, miR-122-5p, miR-24-3p, and miR-4632-5p as a new potential biomarker panel, suggesting to combine gastroscopy with this signature to improve early GC detection. miR-21 levels, in both serum and peripheral blood mononuclear cells, were increased in GC patients compared to controls and could be used in the diagnosis of early (stage I) and late (stage IV) GC^[61]^. In a different work, miR-20a, in combination with miR-1, miR-27a, miR-34, and miR-423-5p, formed a five-miRNA plasma signature for GC detection^[62]^. Serum-elevated miR-378 alone or together with miR-371-5p and miR-187 could be a valuable biomarker to detect early GC^[63]^. Increased miR-515-3p in serum^[64]^ and decreased miR-141^[65]^ in plasma were present in patients with GC compared to healthy controls.

Hung *et al*.^[66]^ found that both plasma and urinary miR-376c were significantly higher in GC patients compared to healthy individuals. Moreover, plasma miR-376c was also validated as biomarker for early stage tumors. In another work, two urinary miRNAs, miR-6807-5p and miR-6856-5p, resulted independent biomarkers for GC diagnosis and the combination of them with the *Helicobacter pylori* status showed excellent power. In addition, this small panel could distinguish between stage I GC patients and healthy controls. Notably, miR-6807-5p and miR-6856-5p urinary levels significantly decreased to undetectable expression after surgery^[67]^.

Li *et al*.^[68]^ identified a signature composed of seven miRNAs (miR-10b, miR-21, miR-223, miR-338, let-7a, miR-30a-5p, and miR-126) able to predict overall and relapse-free survival in GC patients. In a different work, Tsai *et al*.^[69]^ observed an increase in miR-196a and miR-196b preoperative circulating levels in GC patients compared to healthy individuals. Interestingly, the levels of these two miRNAs were reduced after surgery. Moreover, there was a correlation among high circulating miR-196a/b levels and tumor metastatic potential, advanced stages, and poor survival. Finally, they showed that circulating miR-196a and miR-196b, as well as miR-196a and miR-196b combined, succeeded in discriminating GC patients and healthy subjects better than CEA or CA19-9.

Wang *et al*.^[70]^ identified a marked miR-19b and miR-106a overexpression in serum-circulating exosomes in GC patients compared to healthy subjects. Besides, miR-19b and miR-106a expression was correlated with lymphatic metastasis and GC advanced stages (III-IV).

### Glioblastoma

The conventional approaches used for glioma diagnosis and surveillance are Computed Tomography and Magnetic Resonance Imaging, two invasive and quite expensive techniques. There is an urgent need for a new, less invasive way to diagnose and monitor glioblastoma.

Wang *et al*.^[71]^ observed altered miR-21, miR-128, and miR-342-3p levels in the plasma of patients with Glioblastoma Multiforme (GBM) compared to controls. Importantly, this three-miRNA signature did not change in patients with other types of brain tumors, resulting highly specific for GBM. Furthermore, after surgery or chemoradiation, miR-21, miR-128, and miR-342-3p plasma levels normalized and miR-128 and miR-342-3p correlated with histopathological grades of glioma. The importance of miR-21 was underlined also in a meta-analysis by Qu *et al*.^[72]^, who demonstrated high sensitivity and specificity of miR-21 as a single biomarker in glioma diagnosis in EVs. In EVs, miR-451 was also detected as a GBM-associated miRNA^[73]^. A nine-miRNA signature in the CSF correlated with the miRNA profile from tumor tissue and GBM volume^[74]^. In a meta-analysis study analyzing 20 different articles about cell-free microRNAs coming not only from CSF, but also from blood of early glioma diagnosis, cell-free miR-21 resulted as the best miRNA for discriminating glioma patients from healthy individuals, followed by miR-125 and miR-222^[75]^.

### Hepatocellular carcinoma

Hepatocellular carcinoma (HCC) is the major type of primary liver cancer. Alpha-fetoprotein (AFP) was the most widely used HCC biomarker in the past decades. However, AFP shows some limitations; in fact, its increased levels are not detectable in 80% of small HCCs^[76]^. If HCC is diagnosed at < 3 cm, it has over 50% chance of being cured with surgery or thermal ablation^[77]^; thus, it is important to find sensitive biomarkers to detect the pathology at early stage. The expression profile of plasma miRNAs changes more among HCC patients than in healthy individuals^[78]^. As shown by Zhou *et al*.^[79]^, a miRNA panel (miR-21, miR-26a, miR-27a, miR-122, miR-192, miR-223, and miR-801) was able to diagnose hepatitis B virus-related HCC with high accuracy. miR-21, miR-122, and miR-223 were found to be increased in the serum of patients with HCC compared to healthy controls by Xu *et al*.^[80]^, but, unfortunately, also in patients with chronic hepatitis. Thus, these miRNAs may be novel biomarkers for liver injury in general, not specifically for HCC. miR-21 also resulted as a helpful marker for HCC early diagnosis in a meta-analysis by Qu *et al*.^[81]^. As mentioned above, miR-122 levels were higher in HCC patients compared to controls. This result was also confirmed by Qi *et al*.^[82]^, who showed that miR-122 level was significantly reduced in serum samples from patients who underwent surgery compared to their preoperative samples. Tan and colleagues identified a set of eight serum miRNAs (miR-206, miR-141-3p, miR-433-3p, miR-1228-5p, miR-199a-5p, miR-122-5p, miR-192-5p, and miR-26a-5p) characterized by high diagnostic accuracy for HCC and able to discriminate HCC patients from cirrhosis patients and healthy subjects^[83]^.

Serum miR-221, miR-222, miR-21, and miR-224 were differentially overexpressed in HCC patients compared to healthy individuals. Moreover, high miR-221 levels correlated with tumor size, cirrhosis, and tumor stage, resulting important for HCC prognosis^[84]^.

An independent study from Jin *et al*.^[85]^ identified 12 circulating miRNAs differentially expressed between HCC and normal healthy volunteers. Precisely, miR-1972, miR-193a-5p, miR-214-3p, and miR-365a-3p allowed discriminating HCC from non-HCC individuals and six miRNAs emerged as potential prognostic markers for overall survival, with high miR-128, miR-139-5p, miR-382-5p, and miR-410 and low miR-424-5p and miR-101-3p levels in patients with worse survival outcome. In a different study, miR-155 and miR-96 were found upregulated, while miR-99a was downregulated in the serum of HCC individuals; thus, their combination could be a HCC diagnostic biomarker. It is worth noting that AFP measurement, together with the combination of miR-155, miR-96, and miR-99a, resulted highly sensitive and specific for HCC diagnosis compared with a single marker. Moreover, increased miR-155 and miR-96 levels were associated with poor survival in patients with HCC, resulting as potential prognostic markers^[86]^.

Unfortunately, HCC lacks molecular predictors of treatment response. Teufel *et al*.^[87]^ identified nine plasma miRNAs (miR-30a, miR-122, miR-125b, miR-200a, miR-374b, miR-15b, miR-107, miR-320, and miR-645) correlated with overall survival in Regorafenib treated patients.

### Lung cancer

Lung cancer (LC) is the major cause of cancer death worldwide. Nowadays, to diagnose, classify, and set therapy for lung cancer, the molecular characterization of the samples is essential. In particular, EGFR mutations and Anaplastic Lymphoma Kinase translocations are fundamental to decide and administer the therapies to the patient^[88]^. Considering that the five-year survival rate of patients is 13%-15%, it is crucial to find new tools able to detect the cancer at its early stage^[89]^.

Yu *et al*.^[90]^ demonstrated that miR-92a-2 plasma levels were significantly higher in small cell lung cancer (SCLC) patients than in healthy controls, suggesting that it could be a potential biomarker for the diagnosis of SCLC. Shi *et al*.^[91]^ reported that serum levels of miR-125b and miR-22 were significantly increased in patients with non-small cell lung carcinoma (NSCLC) compared to patients with benign lung disease and controls. At the same time, serum miR-15b levels were lower in the patients compared to the other two groups. Notably, serum miR-22 and miR-15b sensitivity in detecting early NSCLC (stage I + II) performed better than CEA. Another study from Heegaard *et al*.^[92]^ observed increased expression of miR-29 and reduced expression of miR-146b, miR-221, let-7a, miR-155, miR-17-5p, miR-27a, and miR-106a in the plasma of NSCLC patients compared to controls. Interestingly, no differences were detected in the plasma of the two groups, showing no correlations of levels between plasma and serum. Conversely, exosomal miR-17-5p expression was significantly upregulated in NSCLC patients compared with healthy controls^[93]^. In a different study, plasma miR-155, together with miR-197 and miR-182, resulted increased in early LC patients (stage I) compared to cancer-free individuals, and miR-155 and miR-197 expression was higher in the plasma from metastatic patients than in those without metastasis and decreased in patients responsive to chemotherapy^[94]^.

Giallombardo *et al*.^[95]^ found an eight-miRNA exosome signature (miR-30b, miR-30c, miR-103, miR-122, miR-195, miR-203, miR-221, and miR-222) that correlated with NSCLC. Later, another exosomal miRNA profile (let-7b, let-7e, miR-23a-3p, and miR-486) allowed distinguishing LC patients from healthy controls. Moreover, exosomal miR-181-5p, miR-30a-3p, miR-30e-3p, and miR-361-5p resulted adenocarcinoma specific, while exosomal miR-10b-5p, miR-15b-5p, and miR-320b squamous cell carcinoma specific^[96]^.

Zhou *et al*.^[97]^ observed a reduction of miR-520f expression in the serum of patients compared with healthy controls and its expression was significantly associated with advanced TNM stage and metastasis. miR-25 resulted important for both diagnosis and prognosis of LC: its plasma expression levels were higher in NSCLC patients compared to controls, and, among the patients, in those with positive lymph node metastasis, poorly differentiation, or advanced clinical stage, it correlated with aggressiveness and poor survival. Moreover, miR-25 and CEA combination could improve the distinction between NSCLC patients and healthy individuals. Notably, miR-25 levels were strongly decreased in patients who underwent surgery^[98]^. miR-25 was also analyzed in a study from Li *et al*.^[99]^ who characterized it in the serum of NSCLC patients and, again, miR-25 expression levels were increased in cancer patients compared to controls and were associated with gender, tumor stage, and lymph node metastasis. miR-25 could even be considered an independent prognostic factor for overall and relapse-free survival. Bianchi *et al*.^[100]^ developed a 34-miRNA panel (miR-test) to classify asymptomatic high-risk individuals with early LC and to discriminate malignant from benign lesions.

### Melanoma

Today, a proper melanoma biomarker does not exist. In fact, tumor histological characteristics such as thickness, ulceration, and mitotic rate are used for staging the pathology^[101]^, but they may be evaluated only after the biopsy. Lactate dehydrogenase (LDH) is the only serum protein biomarker with a prognostic value^[102]^; however, it has several limitations because it is released after cell damage or death and its levels may also become elevated in other tumors^[103]^.

Solé *et al*.^[104]^ identified miR-134-5p and miR-320a-3p as downregulated plasma miRNAs discriminating melanoma patients from healthy subjects. Furthermore, their expression was lower in patients in stage 0 than in healthy individuals and was even reduced in stage I/II patients.

Shiiyama at al.^[105]^ found a panel of serum miRNAs (miR-9-5p, miR-145-5p, miR-150-5p, miR-155-5p, and miR-205-5p) able to discriminate patients with primary tumors from those with metastatic melanoma. miR-150-5p was also deregulated in another study, where Fogli *et al*.^[106]^ investigated the expression of plasma miRNAs in patients at different melanoma stages and found five miRNAs associated with melanoma progression: miR-15b-5p, miR-149-3p, and miR-150-5p resulted upregulated and miR-193a-3p and miR-524-5p downmodulated in patients with melanoma compared with healthy individuals. Greenberg *et al*.^[107]^ reported a strong miR-29c-5p and miR-324-3p downregulation in the serum of metastatic melanoma patients (stage IV) compared to healthy controls. Moreover, miR-29c-5p and miR-324-3p could distinguish among melanoma, colon, and renal cancer patients. Alegre and co-workers demonstrated that miR-125b levels in exosomes were significantly lower in patients with advanced melanoma compared to disease-free subjects and healthy individuals^[108]^. Tengda *et al*.^[109]^ demonstrated that miR-532-5p and miR-106b, isolated from serous exosomes as well as from total serum, were able to discriminate patients with melanoma from healthy controls, metastatic patients from those with no metastasis, patients with stage I-II disease from those with stage III-IV, and patients treated with pembrolizumab from untreated ones.

As mentioned above, miR-150 deregulation is important in melanoma and, together with miR-30d, miR-15b, and miR-425, is part of a four-miRNA signature able to stratify melanoma patients. Moreover, miR-15b levels were increased in recurrent patients compared to non-recurrent ones^[110]^. Decreased miR-206 expression level was detected not only in serum samples from melanoma patients compared to healthy individuals, but also in serum from subjects with metastatic sites and its levels were also associated with the treatment response. The five-year overall and disease-free survival was shorter in melanoma patients with low miR-206 levels compared to patients with high miR-206^[111]^. Stark *et al*.^[112]^ discovered a serum melanoma-related seven-miRNA panel (MELmiR-7) able to discriminate individuals with melanoma from healthy ones with high sensitivity and specificity when approximately four or more miRNAs of the panel were expressed. Interestingly, MELmiR-7 resulted better compared to LDH and S100B in predicting the recurrence and survival of patients. Van Laar *et al*.^[113]^ proposed a bigger signature composed of 38 (MEL38) circulating miRNAs, involved in angiogenesis, invasion, and treatment resistance. MEL38 was able to distinguish melanoma with stage I-IV disease from healthy plasma samples.

Svedman *et al*.^[114]^ characterized EV-miRNAs originating from plasma samples derived from patients before and after therapy. EV let-7g-5p and miR-497-5p levels were increased after Mitogen Activated-Protein Kinase pathway inhibitors, resulting in better disease control and prolonged progression-free survival.

### Ovarian cancer

The first serum biomarker for ovarian cancer (OC), CEA, was discovered in 1965; however, as with other biomarkers, it lacked sensitivity and specificity.

Zhang *et al*.^[115]^ identified miR-106a-5p, let-7d-5p, and miR-93-5p as significantly increased and miR-122-5p, hsa-miR-185-5p, and miR-99b-5p as significantly decreased in the exosomes of patients with OC compared with healthy controls. Similarly, miR-93-5p was increased and miR-99b-5p and miR-122-5p decreased in the plasma of subjects with OC compared to healthy ones. In another study, miR-93, together with miR-145 and miR-200c, was significantly overexpressed in serum exosomes of cancer patients compared to controls. Other miRNAs, such as miR-141, miR-200a, and miR-200b, were expressed at extremely low levels; therefore, they were not as appropriate as serological biomarkers^[116]^. Taylor *et al*.^[117]^ found that eight (miR-21, miR-141, miR-200a, miR-200c, miR-200b, miR-203, miR-205, and miR-214) specific miRNA levels were similar in cellular and exosomal miRNAs and this profile was significantly distinct from what was observed in benign disease. Gong *et al*.^[118]^ demonstrated the prognostic importance of plasma miR-148a-3p; its expression was reduced in OC patients and correlated with histopathologic grade and lymph node metastasis. In fact, higher plasma miR-148a levels were associated with longer overall survival.

Marton *et al*.^[119]^ analyzed nine miRNAs involved in epithelial-mesenchymal transition in the plasma samples of patients with malignant OC, non-malignant OC, or healthy controls. miR-34a, miR-34b, miR-141, miR-200a, miR-200b, miR-200c, miR-203, and miR-429 levels were significantly higher in the malignant samples than in healthy volunteers. Furthermore, miR-141, miR-200a, miR-203a, and miR-429 expression was also higher in malignant compared to non-malignant samples. Similarly, five plasma miRNA (miR-10a-5p, miR-145-5p, miR-205-5p, miR-328-3p, and miR-346) were significantly overexpressed in OC in comparison with normal controls. The same miRNAs were also upregulated in exosomes from plasma patients and plasma miR-205-5p expression may be linked to the histological grade of OC patients^[120]^.

### Pancreatic cancer

Pancreatic cancer (PaC) is a killer cancer because of its poor prognosis, its high metastatic rate, and the absence of early symptoms. In addition, it is difficult to discriminate chronic pancreatitis (CP) patients from PaC ones. Serum CA19-9 allows monitoring patient response to therapy and predict recurrence after surgery, but it has low sensitivity and specificity. Moreover, CA19-9 may be elevated in patients with non-malignant obstructive jaundice and can have normal levels in pre-cancerous masses^[121,122]^.

Liu *et al*.^[123]^ examined serum samples from PaC patients and disease-free controls and observed a seven-miRNA signature (miR-20a, miR-21, miR-24, miR-25, miR-99a, miR-185, and miR-191) able to discriminate various stages of PaC from healthy controls and distinguish PaC patients from those with CP. In detail, serum miR-21 expression levels were significantly associated with overall PaC survival. In a subsequent study, exosomal miR-21 and miR-155 were quantified in the pancreatic juice and compared between Pancreatic Ductal Adenocarcinoma (PDAC), the most diffused kind of PaC, and CP. Relative levels of both exosomal miRNAs, but not free miRNAs, were upregulated in PDAC compared to CP patients and could discriminate PDAC from CP individuals^[124]^. Vila-Navarro *et al*.^[125]^ described let-7e-5p, let-7f-5p, miR-103a-3p, miR-151a-5p, miR-151b, miR-23-3p, miR-320a, miR-33a-3p, miR-548d-3p, and miR-93 circulating miRNAs in PDAC. Furthermore, the miR-33a-3p1 and miR-320a signature could discriminate patients with malignant PaC or premalignant Intraductal Papillary Mucinous Neoplasm from healthy individuals. Notably, the combination of the signature with CA19-9 increased sensitivity and specificity for PDAC early detection. In another study, Zhu *et al*.^[126]^ analyzed plasma samples from PDAC patients and healthy individuals. One hundred sixty-five mature miRNAs resulted differentially expressed between the two groups: 75 were upregulated and 90 downregulated. In particular, the two most upregulated miRNAs in patients were miR-182-5p and miR-4732-5p, whereas the two most downregulated were miR-139-5p and miR-23b-3p.

Interestingly, Cote *et al*.^[24]^ measured not only plasma, but also bile miRNAs to assess a possible differential expression among subjects with PDAC, CP, and controls. A differential expression of nine miRNAs (miR-10b, miR-30c, miR-106b, miR-132, miR-155, miR-181a, miR-181b, miR-196a, and miR-212) in the plasma and seven in the bile (all the ones above excluding miR-132, and miR-181b as well as miR-21) was detected. Among them, miR-10b, miR-155, miR-106b, miR-30c, and miR-212 were accurate for distinguishing PDAC.

### Prostate cancer

Prostate Specific Antigen (PSA) is the election biomarker for prostate cancer (PC), but it shows several limitations: high PSA levels may also be associated with inflamed or enlarged prostate, leading to frequent over-diagnosis. Furthermore, PSA may indicate tumor presence, but it is not able to suggest a proper treatment or give information about tumor recurrence. Importantly, Gleason grade, used to obtain PC prognosis, relies on histological analysis, thus it requires a biopsy, an invasive procedure. miRNAs may be deregulated in the serum of prostate cancer patients versus healthy individuals, such as miR-141, whose level increases in serum of PC patients compared with healthy controls^[11]^. The same miRNA was part of a four-miRNA signature deregulated in the blood PC patients. Precisely, miR-141, miR-145, and miR-155 were upregulated and let-7a was downregulated. Moreover, Kelly *et al*.^[127]^ showed a normalization in miR-141 levels in patients who underwent a radical retropubic-prostatectomy 10 days post-operation. In another study, Matin at al.^[128]^ discovered a four-miRNA panel (miR-98-5p, miR-152-3p, miR-326, and miR-4289) with increased levels in plasma samples from PC patients compared to healthy controls. Mahn *et al*.^[129]^ demonstrated that plasma miR-26a levels allowed discriminating between localized PC from benign prostate hyperplasia. Moreover, Brase *et al*.^[130]^ performed a screening to correlate circulating miRNAs with PC progression. By comparing serum samples from individuals with metastatic and localized tumor, they pinpointed miR-141 and miR-375 as the most pronounced markers for tumor progression, and their release into the blood was associated with advanced cancer. Another group also studied circulating free miRNAs, identifying miR-16, miR-148a, and miR-195 as a unique expression profile for high-risk PC stratification^[131]^. Shen *et al*.^[132]^ demonstrated that the combination of miR-20a, miR-21, miR-145, and miR-221 with high-expression levels in the plasma significantly distinguished intermediate/high-risk from low-risk score patients. Sharova *et al*.^[133]^ demonstrated that miR-106a/miR-130b and miR-106a/miR-223 ratios might discern between patients with localized PC and benign prostatic hyperplasia (BPH). In addition, seven deregulated miRNAs (let-7c, let-7e, let-7i, miR-26a-5p, miR-26b-5p, miR-18b-5p, and miR-25-3p) were able to distinguish between PC and BPH^[134]^. Huang *et al*.^[135]^ identified plasma exosomal miR-1290 and miR-375 as promising prognostic biomarkers for Castration-Resistant PC (CRPC) patients. Some of the above-mentioned miRNAs were part of a signature composed of miR-17, miR-20a, miR-20b, and miR-106a that could discriminate high- and low-risk individuals, in addition to their tumor stage. In particular, high expression of the panel was associated with shorter time to recurrence after radical prostatectomy^[136]^. Moltzahn *et al*.^[137]^ identified a serum miRNA signature for diagnostic and prognostic purpose. In detail, miR-223 decreased, while miR-874 and miR-1207 increased after the transition from healthy to PC. miR-24 continually decreased with risk, while miR-106a increased. miR-26b and miR-30c resulted downregulated in the low- and intermediate-risk groups relative to healthy controls and metastatic cancer. Regarding the response to therapy, serum miR-21 was found elevated in Hormone-Refractory PC patients, in particular in those resulting resistant to docetaxel chemotherapy^[138]^. Lin *et al*.^[139]^ found that high expression of miR-200 and miR-17 families may be early therapeutic response biomarkers to docetaxel treatment in CRPC.

## miRNAs as diagnostic and prognostic markers in hematological malignancies

### Acute myeloid leukemia

Acute myeloid leukemia (AML) is an aggressive hematological disease caused by an abnormal proliferation and differentiation of myeloid progenitor cells^[140]^. It is the most common leukemia in adult patients and it can be *de novo* or secondary^[141]^. Despite a growing list of treatment options, most patients still relapse and die after remission. Currently, cytogenetic markers such as t(8;21), t(15;17), inversion 16, trisomy 8, and deletions of parts, or all, of chromosomes 5 or 7 are used to stratify and treat AML patients. Nevertheless, AML mortality rate is high with a five-year overall survival lower than 50%^[142]^. For these reasons, there is an urgent need for diagnostic and prognostic biomarkers. Reduced expression levels of let-7d, miR-150, miR-339, and miR-342 together with upregulation of let-7b and miR-523 were able to discriminate between AML patients compared to normal controls. Similarly, increased levels of circulating miR-155-3p and miR-181b-5p were present in the blood of AML patients compared to healthy controls^[143]^. In particular, elevated levels of miR-150 and miR-342 after treatment was associated with complete remission in AML patients^[144]^. Moreover, miR-181-5p was associated with shorter overall survival^[143]^. Zhi and colleagues demonstrated that miR-10-5p serum level was higher in *de novo* AML patients compared with healthy controls. As far as AML prognosis is concerned, miR-10-5p expression was significantly higher in relapsed patients compared to patients who underwent complete remission^[145]^. Low miR-210 levels were observed in individuals with complete remission while high miR-210 correlated with poor relapse-free and overall survival^[146]^. High miR-155 expression in AML patients was associated with unfavorable prognosis with lower remission rate and shorter disease-free and overall survival^[147]^. Moreover, Fang *et al*.^[148]^ demonstrated an increase of serum EV-miR-10b in AML patients compared to healthy controls. In addition, its expression was strongly correlated with disease aggressiveness, thus leading to a shorter survival of the patients. In addition, miR-203 is important for both diagnosis and prognosis. In particular, serum miR-203 levels were reduced in AML patients compared with healthy individuals and low miR-203 expression in serum was associated with a decreased overall and relapse-free survival of patients^[149]^.

### Acute lymphoblastic leukemia

Acute lymphoblastic leukemia (ALL) is a hematological malignancy that affects the B (B-ALL) or T (T-ALL) lineages. High hyperdiploidy (51-65 chromosomes) and t(12;21)/*ETV6-RUNX1* are two biomarkers for the diagnosis and prognosis of ALL, in particular for pediatric and adolescent ALL^[150]^. Moreover, chromosomal abnormalities such as *KMT2A* (*MLL*) translocations, t(9;22)/*BCR-ABL1*, t(17;19)/*TCF3-HLF*, near haploidy, and low hypodiploidy are biomarkers able to recognize high-risk disease at all ages^[151]^. Different circulating miRNAs are able to discriminate between B-ALL patients and normal controls; in particular, elevated levels of miR-511, miR-222, and miR-34a were present in the plasma of B-ALL patients. On the contrary, reduced plasma levels of miR-199a-3p, miR-223, miR-221, and miR-26a were observed in B-ALL patients^[152]^. For T-ALL, a rare and aggressive subtype of ALL, few studies are currently available on potential biomarkers. Ishihara and colleagues demonstrated that high expression of miR-155 and low expression of miR-126 in the plasma of T-ALL patients correlated with longer overall survival^[153]^. These two miRNAs performed even better than standard prognostic factors such as LDH and soluble interleukin receptor (sIL-2R). miR-125b-1 expression was increased and miR-203 was decreased in the blood of newly diagnosed children with ALL compared to healthy controls. Interestingly, miR-125-1 was specifically higher in T-ALL compared to other ALL diseases. Importantly, the combination of miR-125b-1 and miR-203 revealed a 100% sensitivity^[154]^.

### Chronic lymphocytic leukemia

Indolent or aggressive Chronic lymphocytic leukemia (CLL) are the most common leukemia, and they are caused by accumulation of incompetent CD5+ B lymphocytes. Nowadays, the Rai and Binet staging systems allow patient stratification into risk groups. Their advantages are their low cost, easy use, and ability to predict overall survival, but they fail in predicting response to treatments. Moreover, chromosome abnormalities such as 17p deletion, 11q deletion, trisomy 12, elevated β2 microglobulin (β2M), thymidine kinase, CD38 expression, unmutated immunoglobulin heavy chain variable gene (IGHV), and ZAP-70 expression are important in poor prognosis prediction^[155]^. miR-155 level in the plasma of CLL patients was useful to predict overall survival; its expression increased with disease progression from monoclonal B-cell-lymphocytosis to CLL; and it was associated with poor response of patients to FCR (fludarabine, cyclophosphamide, and rituximab) chemotherapy. Moreover, miR-155 plasma level was lower in patients who achieved complete remission than in those who experienced other responses^[156]^. miR-155, together with miR-150 and the miR-29 family (miR-29a, miR-29b, and miR-29c), was differentially expressed in the exosomes of CLL patients compared to healthy donors^[157]^. In particular, miR-150 was highly expressed in the serum of CLL patients and was associated with poor prognosis^[158]^. Instead, miR-29 family showed lower expression in a subset of CLL patients and was associated with poor prognosis^[159]^.

### Non-hodgkin lymphoma

Non-hodgkin lymphoma (NHL) consists of a heterogeneous group of lymphoid malignancies (over 50 subtypes) due to altered proliferation of B, T, and natural killer lymphocytes. The most common NHLs are Diffuse Large B-cell lymphoma (DLBCL) and Follicular B-cell Lymphoma (FL)^[160]^. LDH and beta-2 microglobulin (beta 2-M) are prognostic parameters in the staging systems^[161]^. Few studies thus far have attempted to identify circulating miRNA profiles in NHL patients. Increased miR-155, miR-210, and miR-21 levels were observed in the serum of DLBCL patients compared to controls and higher expression of miR-21 was associated with relapse-free survival^[10]^. In another study, DLCBL patients showed high levels of miR-15a, miR-16-1, miR-29c, and miR-155 and decreased miR-34a expression^[162]^. Other circulating miRNAs were differentially expressed in patients who underwent chemotherapy, such as miR-130a and miR-125b, which were upregulated in the drug-resistant group (R-CHOP treatment) compared to the responder group. Moreover, miR-125b was associated with worse prognosis^[163]^. Thus far, no studies have investigated miRNAs as FL biomarkers.

### Multiple myeloma

Multiple myeloma (MM) is characterized by the malignant proliferation of monoclonal plasma cells in the bone marrow and it leads to end stage organ impairment, including bone lesions, renal dysfunction, hypercalcemia, and anemia. Unfortunately, the majority of patients eventually relapse or become refractory following treatment with current therapies. The gold standard for MM diagnosis is bone marrow biopsy, an invasive and painful procedure for patients. Therefore, it is necessary to identify more sensitive, convenient, and noninvasive biomarkers for MM clinical diagnosis. Many different studies have attempted to find circulating miRNAs able to discriminate among MM, the asymptomatic pre-malignant monoclonal gammopathy of undetermined significance (MGUS), and healthy controls. In particular, Jones and colleagues identified six miRNAs (miR-451, miR-638, miR-720, miR-1246, miR-1308, and miR-1915) differentially expressed in MGUS, MM patients, and healthy individuals. More in detail, miR-720 and miR-1308 together were able to discriminate MGUS and MM patients from healthy controls, while miR-1246 and miR-1308 distinguished MGUS and MM patients^[164]^. In the same year, an independent study proposed miR-92a as a MM biomarker able to distinguish healthy controls from MM patients. miR-92a expression changed based on the stage of the disease and the response of patients to therapy^[165]^. A study from Huang and colleagues observed higher expression levels of six miRNAs (miR-148a, miR-181a, miR-20a, miR-221, miR-625, and miR-99b) in the blood of MM patients compared with healthy donors. Among these miRNAs, the levels of miR-99b and miR-221 were associated with t(4;14) translocation and (13q) deletion, while high levels of miR-20a and miR-148a were associated to a shorter relapse-free survival^[166]^. miR-142-5p, miR-660, and miR-29a were upregulated in the serum of MM patients compared to healthy controls^[167]^. Five other miRNAs (miR-744, miR-130a, miR-34a, let-7d, and let-7e) were deregulated in MGUS, newly diagnosed MM, relapsed patients, and healthy controls. In particular, miR-34a and let-7e were able to discriminate MM from healthy patients and MGUS from healthy donors with high sensitivity and specificity. Instead, reduced levels of let-7e and miR-744 correlated with shorter survival and worse time to progression of MM patients^[168]^. Another study described decreased levels of miR-92a, miR-30a, and miR-451 and increased expression of miR-720 in MM patients compared to healthy volunteers. The combination of high serum levels of miR-16 and miR-25 in MM patients correlated with better overall survival if compared with the increased levels of miR-25 alone^[169]^. A panel of four upregulated (miR-1207-5p, miR-3656, miR-630, and miR-483-5p) and eight downregulated (miR-451, miR-92a, miR-22, miR-223, miR-19b, miR-720, miR-16, and miR-20a) miRNAs was able to distinguish between MM patients and healthy controls. Among them, miR-483-5p and miR-20a were both associated with International Staging System, but only miR-483-5p was able to predict progression-free survival^[170]^. As reported above, many studies have been conducted to identify new potential MM biomarkers; some are, at least in part, in agreement, while others are in contrast, e.g., those considering miR-720 and miR-20a. In other studies, single miRNA expression levels were sufficient to predict prognosis, e.g., miR-214, being its high expression detrimental for both progression-free and overall survival^[171]^, and miR-19a, being downregulated in patients with shortened progression-free and overall survival^[172]^. Regarding the identification of biomarkers able to predict the response to therapy in MM, low expression levels of miR-19a in MM patients correlated with a better response and prolonged survival after bortezomib treatment. Similarly, five miRNAs (miR-26a-5p, miR-29c-3p, miR-30b-5p, miR-30c-5p, and miR-331-3p) were downregulated in relapsed/refractory MM patients treated with lenalidomide plus low-dose dexamethasone^[173]^. Five other miRNAs (miR-16, miR-17, miR-19b, miR-20a, and miR-660) were downregulated at the time of diagnosis and increased their levels at complete response after stem-cell transplantation. In particular, miR-19b and miR-331 correlated with longer progression-free survival of the same transplanted patients^[174]^. Finally, circulating miRNAs may also discriminate between patients with bone disease from healthy donors, such as miR-214 and miR-135b^[171]^, or patients with extramedullary disease from healthy donors, as in the case of low miR-130a expression^[175]^.

## Conclusion

Liquid biopsy offers invaluable advantages with respect to classic biopsies: in fact, classic biopsy is an often uncomfortable and sometimes risky procedure. To avoid invasive methods in tumor biopsies, miRNAs in body fluids can serve as liquid biopsy. Despite the high number of proposed miRNA biomarkers for both solid and hematological malignancies, we are still far from their clinical application. Studies on miRNA biomarkers are often single center with a retrospective design. Consequently, many studies do not overlap and are sometimes contradictory. Standardization is still the main issue. The differences observed among the studies are probably due to the choice of the starting material and to variation in the handling of the material, the methodology used, and the internal controls used to normalize data. Sample collection and handling is certainly crucial, but the protocols used for miRNA purification are crucial as well. Moreover, the detection technique together with the lack of standard approach and suitable endogenous reference gene are critical for data reproducibility. Therefore, it is necessary to set up standardized approaches for miRNA biomarker studies. Moreover, it is important to address the question of miRNA specificity. Few reports identify miRNAs specific to one single cancer type: around 55 miRNAs are deregulated in one type of cancer^[176]^. Due to this lack of specificity, the development of a suitable test requires several miRNAs differentially expressed in a specific tumor type. In conclusion, the reports mentioned above highlight the potential of miRNAs in cancer diagnosis, prognosis, and therapy response. However, a huge effort should be made to standardize and optimize protocols.

## Declarations

### Copyright

© The Author(s) 2020.
